# Long-term outcomes after operative versus conservative management of congenital thoracic malformations: a propensity-matched cohort study

**DOI:** 10.1007/s00383-026-06444-0

**Published:** 2026-04-27

**Authors:** Sinja Lambrecht, Julia Elrod, Greta Thater, Meike Weis, Christel Weiß, Christoph Mohr, Laura Wuebken, Michaela Klinke, Johannes Boettcher, Michael Boettcher, Richard Martel

**Affiliations:** 1 Department of Pediatric Surgery, Medical Faculty Mannheim, University Medical Center Mannheim, Heidelberg University, 68167 Mannheim, Germany; 2 Department of Clinical Radiology and Nuclear Medicine, University Medical Center, Heidelberg University, 68167 Mannheim, Germany; 3 Department of Medical Statistics and Biomathematics, Medical Faculty Mannheim, Heidelberg University, Theodor-Kutzer-Ufer 1-3, 68167 Mannheim, Germany; 4 Department of Child and Adolescent Psychiatry, Psychosomatics and Psychotherapy, University Medical Center Hamburg-Eppendorf, 20246 Hamburg, Germany; 5 Department of Pediatric Surgery, University Medical Center Mannheim, Heidelberg University, Theodor-Kutzer-Ufer 1-3, 68167 Mannheim, Germany

**Keywords:** CPAM, Pulmonary sequestration, Hybrid lesion, Lung function, Quality of life

## Abstract

**Purpose:**

Management of asymptomatic congenital thoracic malformations (CTMs) remains controversial. Early resection may prevent infection or malignancy and promote compensatory lung growth, but its long-term impact is unclear. This study compared long-term cardiorespiratory and psychosocial outcomes after operative versus conservative management of CTMs and evaluated open versus thoracoscopic surgery.

**Methods:**

Children with congenital pulmonary airway malformation, pulmonary sequestration, or congenital lobar emphysema treated at a tertiary center between 2000 and 2023 were identified retrospectively. Operative patients underwent open or thoracoscopic resection, while conservatively managed children were followed radiologically. Prospective follow-up included the 6-minute run, pulmonary function testing, and psychosocial assessment. Propensity score matching adjusted for gestational age, birth weight, lesion extent, prenatal intervention, and associated congenital diaphragmatic hernia.

**Results:**

Among 194 children (median follow-up 8.7 years), 162 underwent surgery and 32 were observed. Surgical patients showed smaller prenatal relative lung volume (51% vs. 79%, *p* = 0.02) and higher neonatal acuity (ICU admission 78% vs. 54%, *p* < 0.01). After matching, postnatal length of stay remained longer after surgery (15 vs. 6 days, *p* = 0.03). Thoracoscopy was associated with shorter postoperative stay, fewer complications and no mortality. Long-term fitness and quality of life were comparable between groups (*p* > 0.5).

**Conclusion:**

Surgical management of CTMs does not impair long-term exercise capacity or psychosocial outcomes. Minimally invasive resection appears safe and may be offered as a preventive option in asymptomatic patients.

**Supplementary Information:**

The online version contains supplementary material available at 10.1007/s00383-026-06444-0.

## Introduction

*Congenital thoracic malformations* (CTM) encompass a heterogeneous group of disease entities, such as congenital pulmonary airway malformation (CPAM), pulmonary sequestration (PS), congenital lobar emphysema (CLE) and bronchogenic cyst. CPAM is the most prevalent among these lesions, with an incidence of 1: 11,000–35,000 [9, 17, 37].

Postnatal surgical resection of symptomatic lesions is widely recognized as the treatment of choice [31]. However, the optimal treatment of asymptomatic CPAM remains controversial [31, 33]. The argument for a surgical approach even in asymptomatic CPAM is to prevent recurrent infections which occur in over 30% of conservatively managed patients or malignant transformation in up to 20% of the cases [24]. CPAM Types 1 and 3 represent mosaic-mutations of the Ras/MAPK-pathway (RASopathies) [7, 36].

Thus many advocate resection [31] either open or by thoracoscopy [19]. Current meta-analyses indicate that minimally invasive surgery offers significant advantages in reducing postoperative complications compared to open surgery [19, 38]. Surgery is often advocated because ongoing alveogenesis in the first years of life facilitates compensatory lung growth [30]. Infection rate increases in CPAMs after the second year of life [40]. Performing surgery before infection is associated with lower amount of blood loss and reduced need for lobectomy [40]. Current studies indicate that thoracoscopic resection can be performed at a young age, with minimal associated risks [28, 40, 41].

Data on psychosocial well-being as a long-term outcome in children with congenital thoracic malformations remain limited. Reduced well-being irrespective of disease severity in CDH-patients warrants further investigation into psychosocial outcomes also in CTM [25]. Impaired well-being highlights the need for targeted counselling and psychosocial support.

The objective of this study was to contribute to the ongoing debate regarding conservative versus operative approaches and the feasibility of minimally invasive surgery for CTM. Disease-related measures and clinical outcome were compared between cohorts undergoing open, thoracoscopic or conservative management. A further goal was to prospectively assess long-term psychosocial well-being and cardiorespiratory fitness in children from these cohorts.

## Materials and methods

### Study group

All patients with CTM (treated at the University Medical Center Mannheim, between October 2000 and June 2023 were included. They were categorized into conservative and operative cohorts, the latter comprising an open and a thoracoscopic resection group.

Demographics were assessed including gender, gestational age and birth weight. Lesions were classified according to entity, laterality and affected pulmonary lobes. Perinatal information, relative fetal lung volume (rFLV), presence of polyhydramnios, hydrops fetalis, postnatal mediastinal shift, fetal therapeutic interventions, postnatal length of hospital stay (LOHS), ICU-need, and respiratory support. Additional parameters included preoperative infections, age at surgery, operative time, mortality and synchronous CDH. The postoperative course was characterized by postoperative LOHS, length of ICU-stay, duration of intubation and thoracic drainage, requirement of blood transfusions and postoperative complications (Clavien-Dindo classification).

### Ethics

This study was performed in accordance with the Declaration of Helsinki as revised in 2013 and approved by the ethics committee-II Medical Faculty Mannheim, Heidelberg University (study ID 2022 − 626). For all participants included, informed consent was obtained from the adult participants and for underage patients from the legal guardians. Above the age of six years, age-adapted information was additionally given to the patients and an adapted consent form signed by the patients.

### Therapeutic concept

In symptomatic neonates, resections were performed via thoracotomy. In asymptomatic children, delayed resections by thoracoscopy were recommended - for radiologic correlate of CPAM type 2, the option of conservative management including radiologic follow-up was discussed with the caregivers.

CPAMs and intralobular PS were approached by segmental resection; when the lesion involved hilar structures, lobectomy was performed at the surgeon’s discretion. Atypical resection was performed for peripheral lesions. Unstable patients with PS and HL with severe synchronous malformations (i.e. cardiac malformation, Scimitar syndrome and CDH) were treated by catheter embolization.

### Outcomes

*Lung function*: Spirometry and, from age 9, body plethysmography were used to assess lung function. Vital capacity (VC) indicated restrictive impairment, while the FEV1/FVC ratio (Tiffeneau index) [32] indicated obstructive impairment. Both were measured via volume-flow curves and analyzed using SentrySuite™ software (Version 2, Vyaire Medical). Normal values were determined using the GLI Lung Function Calculator (European Respiratory Center).

*Imaging*: All patients with prenatally diagnosed thoracic malformations underwent intrauterine MRI. Scans were performed with the mother in a supine or mild lateral position, imaging both the lungs and the entire fetus. Due to the high T2 signal of lung parenchyma, HASTE or true FISP sequences were used for lung volumetry; measurements were repeated if motion artifacts occurred [35]. Lung volume was calculated by manual segmentation of lung parenchyma (excluding the hilum) across slices, with volume determined by integrating cross-sectional areas over slice thickness [35]. Relative lung volume (rLV) was calculated by normalizing to expected volumes in a healthy cohort [29]. All asymptomatic patients for delayed resection and conservatively managed patients underwent contrast-enhanced CT between 3 and 6 months of age.

Radiological follow-ups were performed according to the local Standard Operating Procedure: Surgical patients were scanned by a non-contrast CT-scan one year postoperatively to confirm complete resection. Conservatively managed patients had one follow-up CT at the age of two years and MRI scans at the age of six, twelve and eighteen years. Additional radiological follow-ups are based on individual clinical course, presenting symptoms and the results of initial imaging. To minimize the radiation exposure, MRI was applied instead of CT scans from an age of six years whenever feasible. The images were evaluated for mediastinal shifting and presence of recurrence.

All patients were invited to a follow-up survey with evaluation of physical endurance and psychosocial well-being. Non-participation (missing data) was attributable to long distance to the test site, language barriers and outdated contact information.

*Physical endurance*: Physical endurance was assessed with the 6-minute run test (6MR), which is validated for aerobic capacity and is from six years of age (Supplement 1). It was adapted to the local conditions at the institution with a straight 30-meter distance marked as testing course. Participants were instructed to cover the course as often as possible within a 6-minute period. An appropriate pace was demonstrated before testing. The covered distance within the time limit was recorded as an outcome parameter. The results were compared with reference mean values and z-transformed.

*Psychosocial well-being*: Psychosocial well-being was assessed by standardized questionnaires based on age-specific normative data. Tests are summarized in Supplement 1. Data collection was conducted in German using LimeSurvey. Children and caregivers were contacted by telephone, e-mail or post.

### Children’s psychosocial variables

Health-related quality of life was assessed with the Pediatric Quality of Life Inventory 4.0 (PedsQL4.0). Age-adjusted versions were applied for different age categories. From an age of 8 years, children completed self-reports. For younger children, caregivers provided proxy reports.

The Strengths and Difficulties Questionnaire (SDQ) was used as a behavioral screening instrument for mental health beginning from an age of two years. Self-reporting was possible from 12 years of age.

The Parent-Adolescent Communication Scale (PACS) was administered to assess the quality of dyadic parent-child communication as a proxy report from 8 years of age [39]. 

### Caregivers´ psychosocial variables

Parents´ or caregivers´ quality of life was assessed using the EQ-5D-3 L questionnaire, which covers five health dimensions, which are queried on a three-level Likert-Scale. Health status was evaluated on a Visual Analogue Scale (VAS) ranging from 0 (worst) to 100 (best), representing optimal health. A series of three short questionnaires was used to quantify parental mental health, including the Patient Health Questionnaire-9 (PHQ-9), Generalized Anxiety Disorder Scale-7 (GAD-7) and Brief Symptom Inventory-18 (BSI-18). Perceived Stress was measured with the Perceived Stress Scale (PSS-10). Social support was evaluated with the Oslo Social Support Scale 3 (OSSS-3). Socioeconomic status (SES) included the dimensions education, occupational and financial status and was evaluated as an index. These items were assessed with standardized questionnaires. Additionally, the subjective social status was measured with a German version of the MacArthur social scale [10].

### Adult participants

Adult participants completed the same set of standardized questionnaires as parents or caregivers, while the additional basic data corresponded to the children´s survey.

### Statistical analysis

Statistical analysis was performed using SAS Version 9.4 (SAS Institute, Cary, USA). Descriptive variables were reported as median and interquartile ranges (IQR). Binary or categorical variables were presented as absolute and relative frequencies. To analyze differences between the treatment groups (conservative vs. operative; thoracoscopy vs., thoracotomy), chi-square test, Fisher’s exact test, Wilcoxon signed rank test or t-test were used. P-values < 0.05 were considered statistically significant.

To control for known confounding factors, propensity score matching was performed by matching operatively with conservatively managed patients using the procedure PSMATCH with the “region = CS” (common support) option and the “greedy” nearest neighbor matching method. GA, birth weight, affected lobes, prenatal intervention, concomitant CDH were selected as independent variables. A caliper width of 0.25 was specified [27]. Hence, conservatively managed patients were matched with up to two surgically treated cases.

## Results

### Demographics and disease related variables in conservative and operative management

Between October 2000 and June 2023, 194 children were treated at University Medical Center Mannheim for congenital thoracic malformations. Demographics and disease-related characteristics are shown in Table 1. 120 (61.9%) patients were diagnosed with a congenital pulmonary airway malformation (CPAM), 53 (27.3%) pulmonary sequestration (PS), 14 (7.2%) hybrid lesion (HL) and 6 (3.1%) had congenital lobar emphysema (CLE). Six children (3.1%) had other lesions such as bronchogenic cyst, congenital bullae, solitary lung cysts, esophageal duplication cysts, mediastinal teratoma and ectopic fetal liver tissue.

**Table 1.** Demographics and disease related variables in the retrospective cohorts

	Operative (n=162)	Conservative (n=32)	p-value	Surgery: Thoraco-tomy (n=120)	Surgery: thoraco-scopy (n=31)	p-value
Male [%]	57.4	50.0	0.440	55.8	61.3	0.584
Female [%]	42.6	50.0		44.17	38.7	0.686
Gestational age [days]	38+0 *36+5* *-* *39+2*	38+4 *37+5* *-* *39+6*	**0.031**	37+5 *35+7* *-* *38+5*	39+2 *38+1* *-* *39+4*	**<0.001**
Birth weight [g]	3117 *2690 - 3500*	3200 *2950 - 3640*	0.135	3045 *2650 - 3370*	3385 *2935 - 3690*	0.074
Maternal Age [years]	31.7 *28.0 - 34.4*	30.7 *28.6 - 33.3*	0.773	31.5 *27.9 - 34.3*	32.1 *29.0- 35.5*	0.423
Prenatal Data						
CPAM [%]	61.1	65.6	0.631	60.0	67.7	0.430
PS [%]	28.4	21.9	0.449	30.8	16.1	0.103
Hybrid [%]	8.0	3.1	0.473	6.7	9.7	0.697
CLE [%]	2.5	6.3	0.258	2.5	3.2	1.000
Others [%]	3.1	3.1	1.000	1.7	9.7	0.059
Laterality of lesion						
Right [%]	50.9	38.7	0.168	46.2	64.5	0.196
Left [%]	48.5	58.1		52.9	35.5	
Bilateral [%]	0.62	3.2		0.84	0.0	
Affected lobe						
Superior lobe [%]	26.5	25.0	0.856	26.7	32.3	0.536
Inferior lobe [%]	43.2	46.9	0.703	38.3	54.8	0.097
Middle lobe [%]	16.1	3.13	0.054	16.7	12.9	0.785
Several lobes [%]	1.0 *0.0 - 1.0*	1.0 *0.0 - 1.0*	0.453	1.0 *0.0 - 1.0*	1.0 *1.0 - 1.0*	0.105
Lesion volume [ml]	28.1 *9.2 - 58.6*	21.6 *4.3 - 45.0*	0.498	39.1 *9.6 - 59.5*	13.4 *2.2 - 25.5*	0.071
Lung volume [ml]	33.7 *20.4 - 46.5*	42.4 *29.2 - 69.2*	0.104	30.90 *20.4 - 44.9*	38.6 *34.6 - 60.7*	0.226
Relative lung volume [%]	50.8 *32.3 - 71.4*	78.9 *61.6 - 91.5*	**0.015**	46.4 *31.6 - 63.8*	69.7 *51.8 - 110.5*	**0.011**
Lesion/lung volume	0.78 *0.20 - 1.5*	0.57 *0.1 - 1.5*	0.448	0.96 *0.24 - 2.2*	0.51 *0.06 - 0.66*	0.076
CDH [%]	22.2	9.7	0.111	27.5	3.2	**0.004**
Polyhydramnios [%]	17.3	16.1	0.876	20.8	6.5	0.063
Hydrops fetalis [%]	6.2	3.2	0.541	8.3	0.00	0.251
Mediastinal shift [%]	50.6	38.7	0.224	59.2	22.9	**<0.001**
Prenatal Intervention [%]	14.8	6.3	0.262	18.3	6.5	0.166
Perinatal data						
Postnatal respiratory support [%]	62.5	43.8	**0.049**	73.7	32.3	**<.0001**
Postnatal intubation [%]	51.9	12.5	**<.0001**	65.8	9.7	**<.0001**
ECMO [%]	9.3	3.1	0.479	11.7	0.0	0.075
HFOV [%]	12.4	3.1	0.210	15.8	0.0	**0.014**
iNO [%]	22.2	9.4	0.098	29.2	3.2	**0.003**
Surfactant [%]	13.0	3.1	0.135	15.8	3.2	0.077
Postnatal length of hospital stay [days]	22.0 *7.0 - 49.0*	6.0 *3.0 - 9.0*	**<.0001**	29.0 *18.0 - 56.0*	4.0 *3.0 - 6.0*	**<.0001**
Postnatal ICU -need [%]	78.2	53.6	**0.007**	89.3	40.9	**<.0001**

*m*: male, *f:* female, *CS:* Caesarean section, *CPAM:* Congenital pulmonary airway malformation, *PS:* Pulmonary Sequestration, *CLE: *Congenital lobar emphysema, *ICU:* Intensive care unit, *CDH: *Congenital Diaphragmatic Hernia, *ECMO:* Extracorporeal membrane oxygenation, *iNO*: inhaled nitric oxide, *HFOV:* High frequency positive pressure ventilation

Surgical patients were born more prematurely and had smaller relative lung volumes than conservatively managed patients; similarly, patients undergoing open surgery were more premature and had smaller relative lung volumes than those treated thoracoscopically. Initial postnatal care requirements were significantly greater in surgically treated patients overall and, within this group, in those undergoing open surgery. Differences between the sum of both surgical methods and the operative column are explained by the four catheter embolizations and seven operations outside the institution, that were reckoned to the operative group. Data represent medians unless percentage is reported. Interquartile ranges (IQR) are reported in italics. The Chi-square test was applied for dichotomous data, Fisher’s exact test for categorical data and the Wilcoxon signed rank test for non-normally distributed, continuous data.

162 (83.5%) children underwent operative treatment. This figure comprises four (2.1%) catheter embolizations of PS or HL. 32 patients (16.5%) were managed conservatively. Gestational age at birth in the conservatively managed group was only 4 days higher, but statistically significant (*p = 0.031*). The clinical metrics show a significantly shorter postnatal LOHS in the conservative cohort (6 days vs. 22 days; *p*<0.0001). Postnatal ICU-need (78.2% vs. 53.6%; *p* < 0.01), respiratory support (62.5% vs. 43.8%; *p* = 0.049) and intubation (51.9% vs. 12.5%; *p*<0.0001) were significantly more frequent in the operative group. Prenatal MRI findings showed lower rFLV in surgically managed patients (50.8% vs. 78.9%; *p* = 0.015).

### Disease related variables in open and thoracoscopic resection

Among surgically treated children, 120 (74.1%) received an open resection, whereas 31 (19.1%) received a thoracoscopic resection (Table 1). Seven followed-up patients after extrainstitutional operations were not considered in this part of the analysis. Children with open resection had a significantly, however marginally, lower gestational age at birth (264 days vs. 275 days; *p* < 0.001). Postnatal LOHS was 25 days longer for open resection (29.0 days vs. 4.0 days *p*<0.0001) and ICU treatment required more often (89.3% vs. 40.9%; *p*<0.0001) such as respiratory support (73.7% vs. 32.3%; *p*<0.0001). This was reflected by a significantly higher intubation rate and the administration of high frequency positive pressure ventilation and inhaled nitric oxide.

Children with synchronous CDH were more frequently treated by open resection (27.5% vs. 3.2%; *p* = 0.004). Infants with open resection had a lower rFLV in the prenatal MRI (46.4% vs. 69.7%; *p* = 0.011). Postnatal mediastinal shift was significantly more frequent than in children with thoracotomy (59.2% vs.22.6%; *p* < 0.001) however not significantly more than in conservatively managed children (50.6% vs. 38.7%, n.s.).

### Short term postoperative outcome in open and thoracoscopic resection

Open resection as the treatment of choice in neonates was performed at the age of 8 days and thoracoscopic resection at 619 days (*p*<0.0001). The postoperative ICU stay (7 days vs. 1 day IQR; *p*=0.0001) as well as the overall LOHS (21 days vs. 6 days; *p* < 0.0001) were significantly lower after thoracoscopic resection. The persistence of thoracic drainage was one day longer in the open resection group -despite marginal, this was significant (*p* = 0.017). Clavien–Dindo grade ≥ II complications were more frequent after open resection (46.4% vs. 31.0%; *p* < 0.05). Blood products were indicated in 21.1% in the open resection group (*p* = 0.026). Duration of surgery did not differ significantly (open: 95 min; thoracoscopic: 114 min; *p* = 0.15).

### Follow-up imaging data and mortality in operative modalities compared to conservative management

At follow-up, the rate of mediastinal shift had decreased in both conservatively and operatively managed groups (Table 2) compared to postnatal assessments (Table 1.). It was detected in 27% of operatively treated cases and 14% of conservatively managed cases (n.s.). No thoracoscopic patients exhibited mediastinal shift, whereas it was observed in 31% of patients following open resection (*p* = 0.012). In several cases, a reverse shift toward the lesion side occurred post-resection, assessed using the same criteria as mass-effect-related shifts.

**Table 2:** Outcome of operative and conservative management of CTM

	Operative (*n* = 162)	Conservative (*n* = 32)	*p*-value	Surgery: thoraco-tomy (*n* = 120)	Surgery: thoraco-scopy (*n* = 31)	*p*-value
Age at surgery [days]				8.0 4.0–38.5	619 378–1597	**< 0.0001**
Short-term Postoperative Outcome						
Preoperative infections [%]				13.3	25.8	0.103
Duration [minutes]^§^				95 *78–113*	114 *69–172*	0.147
Postoperative ICU-stay [days]^§^				7.0 *3.0–14.0*	1.0 *1.0–2.0*	**0.0001**
Length of hospital stay [days]^§^				21.0 *12.0–40.0*	6.0 *4.0–10.0*	**< 0.0001**
Persistence of thoracic drainage [days]^§^				4.0 *3.0–8.0*	3.0 *1.5–5.0*	**0.017**
Duration of intubation [days]^§^				5.0 *2.5–10.0*	7.0 *4.0–10.0*	0.675
Blood transfusions [%] ^§^				31.4	10.3	**0.026**
Complications [Clavien-Dindo; %] ^§^						
0				17.4	34.5	**0.0495**
I				36.2	34.6	
II				27.5	20.7	
IIIa				5.8	6.9	
IIIb				7. 3	3.5	
Iva				5.8	0.0	
IVb				0.0	0.0	
V				0.0	0.0	
Mortality [%]	4.3	6.3	0.639	4.2	0.00	0.584
Follow-up Cross-sectional Imaging						
Mediastinal shift [%]	26.9	13.8	0.148	31.2	0.00	**0.012**
Recurrence [%]						
Yes	18.5			16.3	21.4	0.896
No	70.8			71.4	71.4	
Unclear	10.8			12.2	7.1	

No significant difference in mortality was observed between the groups, thoracoscopic surgery had favorable outcome regarding mediastinal shift and intensity of postoperative care, keeping an equivalent recurrence rate. Note, thoracoscopic surgery was scheduled at a significantly higher age. Differences between the sum of both surgical methods and the operative column are given by the four catheter embolizations and seven operations outside the institution, that were reckoned to the operative group. Data represent medians unless percentage is reported; interquartile ranges are shown in italics. The Chi-square test was applied for dichotomous data, Fisher’s exact test for categorical data and the Wilcoxon signed rank test for non-normally distributed, continuous data. *ICU* Intensive care unit.

§ Surgeries involving additional procedures were excluded

Freedom from recurrence exceeded 70% in the operative group, with similar rates for open and thoracoscopic approaches. Inconclusive results due to air trapping or scar tissue were noted in 12% of open and 7% of thoracoscopic cases (n.s.), requiring additional follow-up imaging not yet available during the retrospective analysis.

Mortality did not differ significantly between groups and was largely attributable to comorbidities. Among the operative group, two non-survivors had undergone catheter embolization for sequestration: one (29 WG) with severe cardiac malformations, and another with contralateral CDH and embolization-related abdominal ischemia.

In the conservative group, two non-survivors were born before 30 WG: one with concurrent CDH and a relative lung volume (rLV) of 17%, the other with fetal hydrops treated via intrauterine drainage. No deaths occurred in the thoracoscopic subgroup.

### Cardiorespiratory fitness after conservative and operative management

6MR was performed in 23 children. 27 children participated in the assessment of lung function. Physical endurance data showed no significant differences between conservatively and surgically managed children or between open and thoracoscopic resection. For details, see Table 3. VCmax and the Tiffeneau index determined by spirometry along with the 6MR displayed negative z-scores for both groups.

**Table 3:** Long-term fitness parameters in operative and conservative management of CTM

	Operative	Conservative	*p*-value	Surgery: thoracotomy	Surgery: thoracoscopy	*p*-value
Age [years]	8.95 7.66 − 13.74	9.68 7.78 − 12.10	0.680	11.12 7.75 − 15.13	8.95 6.32 − 12.78	0.440
VCmax [z-score]	−1.54 *−2.78* − *−0.67*	−1.79 *−2.50* − *−1.47*	*0.793*	−2.05 *−3.36* − *−0*,*45*	−1.24 *−2.25* − *−0*,*45*	*0.331*
Tiffeneau Index [z-score]	−1.51 *−2.41* − *−0.89*	−0.30 *−1.20* − *- 0.58*	*0.209*	−1.51 *−2.6 - −1.00*	−0.89 *−2*,*41* − *−1.10*	*0.290*
Age [years]	8.60 *7.45 − 13.49*	12.69 *10.03−14.41*	*0.670*	7.84 *7.55 − 14.31*	9.98 *6.52 − 12.33*	*0.630*
6MR [z-score]	−1.49 *−2.01* − *−0.64*	−1.74 *−3.06* − *−1*,*36*	*0.437*	−1.64 *−3.07* − *−0*,*42*	−1.55 *−1.70* − *−0.79*	*0.563*

*VCmax* maximal vital capacity, *6MR* 6 min run test

Equivalent results were measured in spirometry and 6MR comparing operative vs. conservative management as well as open vs. minimally invasive surgery. Data represent medians; interquartile ranges are shown in italics. Differences were evaluated using t-test.

### Psychosocial well-being and socioeconomic data after conservative and operative management

98 patients took part in the survey of quality of life and socioeconomic parameters. No significant differences between conservatively and surgically managed children nor between open and thoracoscopic resection were detected (Table 4). PedsQL [34] and PACS [2] showed high values, indicating good Health-related quality of life and dyadic parent-child communication. SDQ results were within normative ranges [15].

**Table 4:** Quality of live and socioeconomic data

	Operative	Conservative	p-value	Surgery: thoraco-tomy	Surgery: thoraco-scopy	p-value
PedsQL 4.0 [0-100 scale]	83.7 *72.8 - 90.5*	87.1 *76.0 - 91.1*	0.642	83.70 *71.6 - 90.9*	83.70 *79.35 - 88.0*	0.956
*SDQ*	10.0 *5.0 - 14.0*	7.0 *7.0 - 11.0*	0.712	10.0 *5.0-15.0*	11.5 *3.0-13.0*	0.916
*PACS*	90.0 *84.0 - 95.0*	82.0 *79.0 - 89.0*	0.173	90.0 *86.0 - 94.0*	91.0 *85.0 - 95.0*	0.779
EQ5D3L: Index	0.495 *0.260 - 0.495*	0.495 *0.238 - 0.495*	0.532	0.495 *0.238 - 0.495*	0.495 *0.495 - 0.495*	**0.034**
VAS	80.0 *70.0-90.0*	80.0 *72.0-84.5*	0.800	80.0 *68.0-90.0*	80.0 *71.0-90.0*	0.816
PHQ-9	3.0 *2.0-6.0*	4.5 *3.0-5.5*	0.402	3.0 *2.0-6.0*	4.0 *1.0-8.0*	0.677
GAD-7	4.0 *2.0-6.0*	3.5 *1.5-5.5*	0.754	4.0 *2.0-6.0*	4.0 *1.0-7.0*	0.595
BSI-18	3.0 *1.0-6.0*	2.5 *1.0-5.0*	0.716	3.0 *1.0-5.0*	2.0 *0.0-7.0*	0.969
OSSS-3	11.0 *10.0-12.0*	10.5 *8.5-12.0*	0.399	11.0 *9.0-12.0*	11.0 *10.0-13.0*	0.349
PSS-10	13.0 *9.0-17.0*	13.5 *9.0-18.0*	0.960	12.0 *9.0-18.5*	14.0 *8.0-16.0*	0.698
SES Index: Total	14.2 *12.2-17.7*	15.6 *12.6-17.9*	0.865	14.6 *12.2-17.7*	13.2 *12.4-15.4*	0.680
SES Index: Education	3.6 *3.0-6.7*	3.6 *3.3-6.9*	0.421	3.6 *3.0-6.7*	3.6 *3.0-7.0*	0.866
SES Index: Occupation	4.7 *4.4-5.6*	4.7 *4.4-5.6*	0.890	4.7 *4.4-5.6*	4.7 *4.4-5.4*	0.226
SES Index: Financial	5.0 *4.0-6.0*	5.5 *5.0-6.5*	0.329	5.0 *4.0-6.0*	5.3 *3.0-6.5*	0.932
Mac Arthur Social Scale	6.0 *5.0-7.0*	7.0 *6.0-7.0*	0.514	6.0 *5.0-7.0*	6.0 *5.0-7.0*	0.612

*PedsQL*:Pediatrics Quality of Life Inventory, *SDQ:* Strength and Difficulties Questionnaire, *PACS:* Parent Adolescent Communication Scale, *PSS 10:* Perceived Stress Scale 10,* EQ5D3L:* European Quality of Life 5 Dimensions 3 Level Version, *VAS:* Visual analog scale, *PHQ9:* Patients Health Questionnaire 9, *GAD7:* Generalized Anxiety Disorder 7, *BSI 18:* Brief Symptom Inventory 18, *OSSS3:* Oslo Social Support Scale 3, *SES:* socioeconomic status,

Quality of life, as assessed by the EQ-5D-3L index, was higher in patients undergoing thoracoscopic resection than in those treated with open surgery. Apart from this difference, no significant differences were observed between surgical and conservative management or between open and thoracoscopic approaches. Data represent medians unless percentage is reported; interquartile ranges are shown in italics. Significance of differences were assessed by the Wilcoxon signed rank test.

§§: Classification according to treatment at time of evaluation, i.e. five patients from table 1-3 were operated after the survey.

Caregiver´s depression levels measured by PHQ-9 ranged between minimal to moderate [16] and anxiety levels (GAD-7) indicated minimal burden. BSI-18 values aligned German normative population. PSS-10 values reflected normal stress levels with relatively high self-efficacy [14]. EQ-5D-3 L indices presented generally high values. The EQ-5D-3 L was significantly higher for caregivers of the thoracoscopic resection group (open: 0.495, IQR [0.238 − 0.495]; thoracoscopic: 0.495 IQR [0.495 − 0.495]; *p* = 0.034). Social support according to OSSS-3 was at an intermediate level [12].

Since the open resection group contained adult patients, a comparison of outcome parameters was not possible for this cohort. GAD7 and PHQ9 [16] suggested minimal burden. BSI-18 values were within the range of the norm. PSS-10 values for helplessness ranked within the range of a German community sample, whereas self-efficacy reached higher values [14]. The EQ-5D-3 L indices showed generally high levels. Social support could be classified as strong [12].

Socioeconomic status was largely in the upper two quintiles.

### Propensity score matching for lesion parameters and short-term postoperative outcome

To control for known confounding factors, propensity score matching for GA, birth weight, affected lobes, prenatal intervention and concomitant CDH was performed. Up to two surgically treated patients were matched to every conservatively managed patient, resulting in 26 patients in the conservative and 51 in the operative group. Testing the postoperative outcome and disease-related data of these groups showed only a significantly longer postnatal LOHS in surgically managed children (14.5 days vs. 6; *p* < 0.05; Table 5).

**Table 5:** Lesion parameters and short term perinatal outcome after propensity score matching

	Operative (*n* = 51)	Conservative (*n* = 26)	*p*-value
Length of hospital stay [days]	14.5 4.0–23.0	6.0 5.0–9.0	0.0496
ICU-need [%]	64.6	57.7	0.560
Mortality [%]	1.96	7.69	0.262
Lesion volume [ml]	11.5 *5.3–33.2*	21.6 *4.3–45.0*	0.606
Lung volume [ml]	42.3 *31.0–64.6*	43.4 *29.2–69.2*	0.858
Relative lung volume [%]	66.5 *51.3–110.5*	78.9 *61.6–91.5*	0.733
Lesion/lung volume	0.34 *0.08–1.20*	0.57 *0.09–1.53*	0.635

*ICU*Intensive care unit

After matching, the difference in length of hospital stay comparing surgical and conservative patients became less, previously significant differences in terms of relative lung volume and overall ICU-need were no longer significant. Differences were calculated using chi-square or Wilcoxon rank test. Data represent medians unless percentage is reported. Interquartile ranges are set in italics.

### Propensity score matching for psychosocial well-being

Propensity score matching was performed as well for the outcome of psychosocial well-being, using identical matching parameters. This resulted in a group of 29 conservatively and 52 surgically managed patients (at the time-point of the survey for psychosocial parameters). No significant differences were detected between the cohorts (Table 6).

**Table 6:** Quality of live and psychosocial data after propensity score matching

	Operative (*n* = 52)	Conservative (*n* = 29)	*p*-value
Childrens´ QoL and psychosocial stress			
PedsQL 4.0	81.7 *72.8–91.3*	88.1 *81.5–91.7*	0.376
SDQ	11.0 *3.5–16.5*	7.0 *6.0–11.0*	0.256
Caregivers’ QoL and psychosocial stress			
EQ-5D-3 L: Index	0.50 *0.28–0.50*	0.50 *0.28–0.50*	0.951
VAS	81.0 *75.0–90.0*	79.0 *70.0–85.0*	0.301
PHQ-9	3.0 *2.0–6.0*	4.0 *3.0–7.0*	0.500
GAD-7	4.0 *4.0–6.0*	2.0 *0.0–5.0*	0.477
BSI-18	3.0 *1.0–6.0*	3.0 *0.0–5.0*	0.652
PSS-10	14.0 *10.0–19.0*	12.0 *9.0–18.0*	0.544

*PedsQL 4.0* : Pediatrics Quality of Life Inventory 4.0, *SDQ:* Strength and Difficulties Questionnaire, *EQ-5D-3 L:* European Quality of Life 5 Dimensions 3 Level Version, *VAS:* Visual analog scale, *PHQ-9:* Patients Health Questionnaire 9, *GAD-7:* Generalized Anxiety Disorder 7, *BSI-18:* Brief Symptom Inventory 18, *PSS-10:* Perceived Stress Scale 10. Differences were calculated using

After matching, no significant differences between surgical and conservative patients are revealed. Data represent medians unless percentage is reported; interquartile ranges are shown in italics.

## Discussion

With a median follow-up of 8.7 years, this single-center cohort provides a comprehensive appraisal of both functional capacity and psychosocial well-being after different management strategies for congenital thoracic malformations (CTM). After adjustment for key perinatal confounders, we found no clinically relevant differences in adolescent exercise performance, lung-function indices or quality-of-life scores between children who underwent early resection and those who were managed expectantly. These findings indicate that surgery, when indicated, including thoracoscopic segment- or lobectomy, does not incur a long-term physiological or psychosocial penalty, while it does remove the recognized downstream hazards of infection and malignant transformation. Given potential diagnostic inaccuracy, the risk of malignancy is estimated at 4% for pleuropulmonary blastoma and an additional undefined risk for late malignant transformation according to systematically reviewed literature [5].

Patients selected for surgery had significantly higher neonatal acuity and smaller prenatal relative lung volumes which may have guided the treatment decision. They displayed greater initial morbidity, reflected by longer neonatal hospitalization and higher requirements for ventilation and intensive care. Propensity-score matching for gestational age, birth weight, lesion extent, prenatal intervention and synchronous CDH neutralized most of these disparities, leaving only a modest (~ 9-day) prolongation of postnatal stay in the surgical cohort. This attenuation underlines that the decision to operate is driven less by surgeon preference and more by lesion biology and perinatal compromise, a conclusion consonant with recent multicenter data showing that one in five “high-risk” malformations can nonetheless be deferred safely to later infancy [13, 21].

The striking age difference between open (median 8 days) and thoracoscopic (median 20 months) procedures reflects prevailing technical limits in the early 2000s. These findings also underscore the clinical reality that large pulmonary malformations may cause significant neonatal respiratory compromise, necessitating early surgical intervention and ruling out both an initial conservative strategy and a delayed thoracoscopic approach [11, 22]. Nevertheless, whenever anatomy and cardiorespiratory stability allowed a minimally invasive approach, thoracoscopy halved hospital and ICU stay, reduced complication and transfusion rates, and caused no deaths. These findings are consistent with those of a meta-analysis by Adams et al. [1], which reported reduced postoperative LOHS and complication rates following thoracoscopic surgery. In contrast to Adams et al., who observed significantly longer operative times in thoracoscopic procedures, the data of the present cohort did not show any differences [1, 11, 22, 41].

These advantages reflect contemporary studies that favor thoracoscopy for both symptomatic and prophylactic resections [6, 26]. Evidence reviewed by the American Pediatric Surgical Association (APSA) committee [5] supports early resection (1) to allow for optimal potential for compensatory lung growth and (2) prior to the onset of symptoms. The conclusion of the systematically reviewed literature did not clearly define an optimal age at which the balance between operative risk and pulmonary growth is maximized, however, the data suggested that intervention within the first six months may offer technical advantages, enable smoother recovery, and still allow sufficient opportunity for compensatory lung development [5].

Follow-up imaging confirmed freedom from recurrence in over 70% of operatively treated patients, with comparable rates for open and thoracoscopic approaches. However, approximately 11% of cases yielded inconclusive imaging findings attributable to air trapping or postoperative scar tissue, highlighting the interpretive challenges inherent to post-resection surveillance and the potential need for multimodal imaging strategies at longer follow-up intervals.

### Cardiorespiratory fitness

Both operative and conservative cohorts exhibited mildly negative z-scores for vital capacity and FEV₁/FVC, suggesting a small but persistent decrement in absolute lung volume. The absence of an inter-group gradient implies that this reduction reflects the congenital deficit (or resected parenchyma). Elevated indices suggestive of airway obstruction could reflect mechanical compression from residual lesions in conservatively managed children or structural distortion of the airways resulting from surgery [8]. Although continued alveolarization in early childhood offers a window for catch-up growth [30], the present data and previous reports indicate that compensatory lung growth may be incomplete, underscoring the need for long-term functional surveillance regardless of treatment modality [23].

Importantly, the observation that both groups displayed mildly reduced spirometric values relative to population norms suggests an intrinsic, disease-related limitation that is independent of the management strategy chosen. From a clinical perspective, these decrements are modest and did not translate into impaired exercise performance; nevertheless, they support the recommendation for structured pulmonary follow-up into adolescence and adulthood, regardless of whether resection was performed.

Lau et al. [18] found significantly better FVC and FEV₁ values in favor of the minimally invasive approach, supporting the potential functional advantage of minimal invasiveness. Because our center adopted the minimally invasive approach only recently—particularly for neonates—ongoing follow-up may yet reveal further benefits for the (minimal-invasive) approach.

### Long-term psychosocial well-being

The psychosocial stress as a long-term outcome parameter following resection of congenital thoracic malformations has been largely uncharted. In the present cohort, both patients and caregivers—across the conservative and operative groups—demonstrated high levels of health-related quality of life and only mild to moderate psychosocial stress. No statistically significant differences were observed between the two management strategies in these outcomes, indicating a generally favorable long-term psychosocial profile regardless of treatment modality. These findings are consistent with the results reported by Nuutinen et al. which represent the very scarce data about this topic [23]. Nuutinen et al. compared PedsQL scores between children surgically treated for congenital pulmonary airway malformation (CPAM) and healthy controls, and similarly found no significant differences [23].

Despite the overall reassuring group-level results, individual cases exhibited impaired psychological well-being. These findings are consistent with previous reports, indicating that psychosocial well-being is independent of disease severity [3, 25]. It highlights the need for a comprehensive and ongoing follow-up that includes systematic assessment of psychosocial well-being for both operatively and conservatively managed patients and their families [20]. This may enable healthcare providers to identify and intervene on psychosocial concerns [20]. Continuous psychological support and counseling should be offered concerning the parent–child interaction [4].

### Limitations

This study is limited by its retrospective design for peri-operative data and potential selection bias in surgical allocation. Additionally, participation in the prospective fitness assessment was low, limiting the representativeness of this subgroup; therefore, findings related to fitness should be considered exploratory. A clear selection bias exists in the choice of surgical approach: neonates with respiratory distress were more likely to undergo open resection, whereas clinically stable patients were either managed conservatively or received delayed minimally invasive surgery. The absence of randomization further limits the direct comparability of treatment groups. To mitigate the influence of confounding variables particularly those related to perinatal morbidity, we employed propensity score matching. While this method improves group comparability, it cannot fully substitute for randomized controlled data.

The inclusion of eleven patients treated partly outside our institution (seven after extrainstitutional surgery, four after catheter embolization) may introduce heterogeneity and potential bias. These cases were retained to avoid selection bias and to preserve relevant functional follow-up data. In a post-hoc sensitivity analysis excluding these eleven patients (*n* = 183), all principal comparisons between operative and conservative management remained unchanged: quality-of-life scores (PedsQL Total, *p* = 0.20), spirometric indices (VC z-score, *p* = 0.53; Tiffeneau z-score, *p* = 0.53) and exercise capacity (6MR z-score, *p* = 0.95) showed no significant inter-group differences.

Moreover, given the duration of follow-up, this study cannot provide insight into the risk of malignant transformation. Accordingly, substantially longer-term data will be required to adequately address this important question.

In the prospective portion of the study, participation was voluntary, and the response rate, especially for assessments of cardiorespiratory fitness, was relatively low. As such, participation bias cannot be excluded. The absence of a contemporaneous healthy control group mitigated only partly by normative z-scoring, constrains interpretation of subtle functional decrements. Finally, refining surgical techniques as well as considerably evolving neonatal critical care and ventilation strategies over the 23-year span may blur temporal comparisons. The proportion of patients managed operatively declined gradually from 100% in the earliest birth cohorts (before 2004) through 85% (2011) with a nadir of 67% in 2013 and subsequently increased again to 80% for the patients born in 2023, reflecting the evolving debate on indications for resection.

## Conclusion

In this 23-year propensity-matched cohort, children with congenital thoracic malformations managed operatively or conservatively showed no relevant differences in long-term exercise performance, lung function, or psychosocial well-being. Thoracoscopic resection was associated with shorter hospitalization and fewer perioperative complications. In light of this, clinicians may counsel families that prophylactic minimal-invasive resection does not compromise long-term fitness or quality of life, while offering protection against infectious and oncologic complications.

## Supplementary Information

Below is the link to the electronic supplementary material.

Supplementary Material 1

## Data Availability

The data supporting the findings of this study are not publicly available due to their sensitive nature, arising from the rarity of the congenital condition and the associated risk of patient identification, as assessed by the ethics committee. The data are held in controlled-access storage at the University Medical Center Mannheim and may be obtained from the corresponding author upon reasonable request.

## Abbreviations

6MR: 6 Minutes run test
BSI 18: Brief symptom inventory
CDH: Congenital diaphragmatic hernia
CTM: Congenital thoracic malformations
CPAM: Congenital pulmonary airway malformation
CT: Computed tomography
ECMO: Extracorporeal membrane oxygenation
FEV1: First second of forced expiration
FVC: Forced vital capacity
GAD 7: General anxiety disorder scale
GMT: German motoric test
HL: Hybrid lesion
ICU: Intensive care unit
IQR: Interquartile range
LOHS: Length of hospital stay
MRI: Magnetic resonance imaging
OSSS 3: Oslo social support scale
PACS: Parent-adolescent communication scale
PHQ 9: Patient health questionnaire
PS: Pulmonary sequestration
PSS 10: Perceived stress scale
rFLV: Relative fetal lung volume
SES: Socioeconomic status
SDQ: Strength and difficulties questionnaire
VAS: Visual analogue scale
VCmax: Maximum vital capacity
