# Supplementary material for: Long-term outcomes after operative versus conservative management of congenital thoracic malformations: a propensity-matched cohort study
Source: Pediatr Surg Int. 2026 Apr 27;42(1):209. doi: 10.1007/s00383-026-06444-0 (PMC13121396; doi:10.1007/s00383-026-06444-0)
Supplement: Supplementary file 1 — Supplementary Material 1 [file 383_2026_6444_MOESM1_ESM.docx]

Supplement 1/1, Pediatric Surgery International

Manuscript title: Long-term outcomes after operative versus conservative management
of congenital thoracic malformations: A propensity-matched cohort study

Lambrecht S^1^, Elrod J^1^, Thater G^2^, Weis M^2^, Weiß C^3^, Mohr C^1^, Wuebken L^1^, Klinke M^1^, Boettcher J^4^, Boettcher M^1^, Martel R^1^

1 Department of Pediatric Surgery, University Medical Center Mannheim, Medical Faculty Mannheim, Heidelberg University, Germany

2 Department of Clinical Radiology and Nuclear Medicine, University Medical Center Mannheim, Heidelberg University

3 Department of Medical Statistics and Biomathematics, Medical Faculty Mannheim, Heidelberg University, Theodor-Kutzer-Ufer 1-3, Mannheim, Germany

4 Department of Child and Adolescent Psychiatry, Psychosomatics and Psychotherapy, University Medical Center Hamburg-Eppendorf, 20246, Hamburg, Germany

Corresponding author: Richard D. Martel, M.D. E-Mail: richard.martel@medma.uni-heidelberg.de

| **Acronym** | **Nomenclature** | **Description** | **Reference** |
| --- | --- | --- | --- |
| *Physical endurance:* | |  |  |
| 6MR (GMT) | 6-minute run test, element of the German Motoric Test | physical endurance | Bös K, Schlenker L, Büsch D, Eberhardt T, Müller H, Niessner C, Tittelbach S, Woll A. 2023. Deutscher Motorik-Test 6-18. Hamburg: Feldhaus. |
|  |  |  | von Haaren-Mack B, Haertel S, Seidel I, Schlenker L, Boes K. 2011. Die Validität des 6-Minuten-Laufs und 20m Shuttle Runs bei 9-bis 11-jährigen Kindern. Deutsche Zeitschrift für Sportmedizin. 62(11):351-355. |
|  |  |  |  |
| *Children´s Psychosocial Variables* | |  |  |
| PedsQL4.0 | Health related quality of life (HRQoL) |  | Varni JW, Seid M, Kurtin PS. 2001. Pedsql™ 4.0: Reliability and validity of the pediatric quality of life inventory™ version 4.0 generic core scales in healthy and patient populations. Medical care. 39(8):800-812. |
| SDQ | Strengths and Difficulties Questionnaire | mental health | Koglin U, Barquero B, Mayer H, Scheithauer H, Petermann F. 2007. Deutsche Version des Strengths and Difficulties Questionnaire (sdq-deu). Diagnostica. 53(4):175-183. |
|  |  |  | Lohbeck A, Schultheiß J, Petermann F, Petermann U. 2015. Die deutsche Selbstbeurteilungsversion des Strengths and Difficulties Questionnaire (sdq-deu-s). Diagnostica |
| PACS | Parent-Adolescent Communication Scale | quality of dyadic parent-child communication | Zapf H, Boettcher J. 2025. Psychometric evaluation of the german version of the parent-adolescent communication scale. European Child & Adolescent Psychiatry. 34(3):1097-1109. |
|  |  |  |  |
| *Caregivers´ Psychosocial Variables* | |  |  |
| EQ-5D-3L | European Quality of Life 5 Dimensions 3 Level Version | parents´ or caregivers´ quality of life | EuroQol Research Foundation 2018. Eq-5d-3l user guide. p. https://euroqol.org/publications/user-guides. |
|  |  |  | The EuroQol Group 1990. Euroqol-a new facility for the measurement of health-related quality of life. Health policy. 16(3):199-208. |
| GAD-7 | Generalized Anxiety Disorder Scale-7 | signs of generalized anxiety disorder | Spitzer RL, Kroenke K, Williams JB, Lowe B. 2006. A brief measure for assessing generalized anxiety disorder: The gad-7. Arch Intern Med. 166(10):1092-1097. |
| PHQ-9 | Patient Health Questionnaire-9 | parental mental health | Kroenke K, Spitzer RL, Williams JB. 2001. The phq-9: Validity of a brief depression severity measure. J Gen Intern Med. 16(9):606-613. |
| BSI-18 | Brief Symptom Inventory-18 |  | Spitzer C, Hammer S, Lowe B, Grabe HJ, Barnow S, Rose M, Wingenfeld K, Freyberger HJ, Franke GH. 2011. [the short version of the brief symptom inventory (bsi -18): Preliminary psychometric properties of the german translation]. Fortschr Neurol Psychiatr. 79(9):517-523. |
| PSS-10 | Perceived Stress Scale-10 | perceived distress | Schneider EE, Schonfelder S, Domke-Wolf M, Wessa M. 2020. Measuring stress in clinical and nonclinical subjects using a german adaptation of the perceived stress scale. Int J Clin Health Psychol. 20(2):173-181. |
| OSSS-3 | Oslo Social Support Scale 3 | social support | Dalgard OS, Dowrick C, Lehtinen V, Vazquez-Barquero JL, Casey P, Wilkinson G, Ayuso-Mateos JL, Page H, Dunn G, Group O. 2006. Negative life events, social support and gender difference in depression: A multinational community survey with data from the odin study. Social psychiatry and psychiatric epidemiology. 41:444-451. |
| SES | Socioeconomic Status Scale | educational, occupational and financial status | Lampert T, Müters S, Stolzenberg H, Kroll LE. 2014. Messung des sozioökonomischen status in der kiggs-studie. |
|  |  |  | Müters S, Michalski N, Hoebel J. 2023. Aktualisierung der berechnungsgrundlagen für den index des sozioökonomischen status in der studie gesundheit in deutschland aktuell (geda) 2019/2020-ehis. |
|  |  |  | Lampert T, Hoebel J, Kuntz B, Müters S, Kroll LE. 2018. Messung des sozioökonomischen Status und des subjektiven sozialen Status in Kiggs Welle 2. |
| MacArthur SSS Scale | MacArthur Scale of Subjective Social Status | person’s perceived rank relative to others | Hoebel J, Müters S, Kuntz B, Lange C, Lampert T. 2015. Messung des subjektiven sozialen Status in der Gesundheitsforschung mit einer deutschen Version der MacArthur Scale. |

**Supplement 1:** List of applied physical and psychosocial tests accompanying the follow-up of patients with operative and conservative management of congenital lung lesions.
